# Quantitative analysis of age‐related changes in vascular structure, oxygen saturation, and epidermal melanin structure using photoacoustic methods

**DOI:** 10.1111/srt.13537

**Published:** 2024-01-04

**Authors:** Koji Mizukoshi, Hideaki Iwazaki, Taiichiro Ida

**Affiliations:** ^1^ POLA Chemical Industries, Inc. Yokohama Kanagawa Japan; ^2^ Advantest Corporation Saitama Japan

**Keywords:** melanin condition, oxygen saturation, photoacoustic measurement, vascular structure

## Abstract

**Background:**

Vascular structure, blood oxygen saturation, and melanin status of the epidermis are chromophore factors related to light absorption. Therefore, they are likely to be related to skin appearance. Thus, it is important to measure these internal skin features and understand their characteristics. Thus, we aimed to analyze the individual differences and aging changes in the skin by measuring the internal skin characteristics, such as vascular structure, oxygen saturation, and the 3D distribution of melanin in the epidermis, using a noninvasive photoacoustic (PA) measurement method.

**Materials and Methods:**

A PA measurement device was used as a noninvasive measurement method. Eighty Japanese women aged between 20 and 60 years were enrolled. The target area was the buccal region of the face.

**Results:**

The blood vessel structure showed a decrease in fine vessels with age, with a stronger tendency observed in the dermis layer, and the volume of blood vessels was larger in the dermis layer than in the dermal–subcutaneous fat boundary layer. Oxygen saturation showed a similar decreasing trend with age in all depths examined. Melanin condition as the torus‐like pattern structure tended to increase with age.

**Conclusion:**

PA measurements revealed the characteristics of several chromophores, providing a new skin aging mechanism.

## 1 INTRODUCTION

The skin has various internal structural features that affect various skin functions and skin surface feature such as color. Therefore, for cosmetic/aesthetic purposes, it is important to measure these internal skin features with an accuracy that allows detection of aging changes and individual differences. Particularly, vascular structure, blood oxygen saturation, and epidermal melanin are chromophore elements^1^ related to light absorption and are likely to be related to skin appearance in terms of color. Therefore, it is important to measure and understand these internal skin characteristics. Additionally, noninvasive measurement methods are needed for studies on facial skin, as invasive measurements are difficult to perform on a large number of specimens from the viewpoint of obtaining samples.

As noninvasive skin measurement methods, various techniques utilizing physical phenomena, such as ultrasound and various types of light, have been constructed and used for measurement. Ultrasound has relatively low scattering and low attenuation in tissues, allowing measurements to be taken deeper into the skin. Therefore, skin thickness and density have been measured at depths that reach into the subcutaneous fat layer.^2^ Contrarily, the low absorption specificity of tissues make it difficult to obtain detailed features other than thickness and density, making the measurements of vascular structure, blood oxygen saturation, and melanin distribution difficult. Optical techniques, including Raman spectroscopy,^3^ multiphoton^4^ and Second harmonic generation (SHG),^5^ Optical Coherence Tomography (OCT),^6^, ^7^, ^8^ and in vivo confocal laser microscopy^9^ can be used to make factor‐specific measurements inside the skin due to their high absorption specificity. However, these conventional methods have not been able to measure the deeper layers of the skin due to light attenuation and have been used to measure the structure of the shallow layers of the skin, such as the papillary dermis layer. Therefore, the characteristics of the vascular structure in the shallow skin layer have been investigated using conventional measurement methods,^10^ but deeper layers have not been examined. Our previous NIRS study^11^ reported on the oxygen saturation of the entire skin layer, but it has not examined the characteristics at different skin depths or in relation to the vascular structure.

The epidermal melanin distribution on age spots, where melanin granules, are dense has been measured by conventional measurement methods.^9^ Contrarily, the distribution of melanin in normal skin, where melanin granules are not dense, has not been measured because it has not been possible to visualize melanin appropriately. Uneven coloration tends to occur with aging even in areas other than age spots, and changes in the distribution state of melanin are considered.^12^, ^13^ More recently, the amount of melanin distribution in normal skin was examined using an in vivo multiphoton measurement system.^4^ However, the 3D shape of melanin distribution state of melanin at depths from the stratum corneum to the epidermal basal layer in terms of individual differences and aging changes has not been examined, and it is necessary to introduce a method that can measure it.

A noninvasive skin measurement method is a new perspective that combines the advantages of the abovementioned conventional measurement methods. This technique is the photoacoustic (PA) method, which combines the advantages of the absorption specificity of light and the low attenuation of sound.^14^ The PA method is a phenomenon in which chromophores are exited selectively by pulsed light and emit thermoelastic waves through adiabatic expansion.

This phenomenon allows us to measure specifically and deeply into the skin for features, such as blood vessels, blood oxygen saturation, and melanin, which are light‐absorbing substances. The method has been studied for melanoma^15^ and other skin diseases,^16^, ^17^ but has not yet been studied for normal subjects. In this study, we aimed to analyze the individual differences and aging changes in the normal healthy skin by measuring the internal skin characteristics, such as vascular structure, oxygen saturation, and the 3D distribution of melanin in the epidermis, using a noninvasive PA measurement method.

## 2 METHODS

### 2.1 Study participants and measurement area

Altogether, 80 Japanese women aged between 20 and 60 years were assigned equally in terms of age. This determination was based on calculations performed using G*Power,^18^ where an effect size of 0.3 in the correlation analysis necessitated at least 64 subjects to ensure adequate statistical power and validity in the findings.

Written informed consent forms were obtained from all participants prior to study participants. The study was approved by the Ethics Committee of POLA Chemical Industries. The targeted measurement area was the left cheek; the center point of measurement was the intersection of a perpendicular line extending from the external eye corner to the face line and a horizontal line extending from the nose apex to the ears. Measurements were taken in the supine position with the face oriented in a direction wherein the area including the center point of the measurement was generally horizontal. If a spot was visually observed on the cheek, it was avoided and the measurement was performed on a normal area in the vicinity.

### 2.2 Microscope measurement

Skin surface image measurements were taken with an in‐house video microscope with the area to be measured as the center of the image. The following two types of images were obtained from the same area using a polarization filter: a normal reflection image and a normal reflection eliminated image. The optical magnification was 30×.

### 2.3 Photoacoustic measurement

**FIGURE 1:**
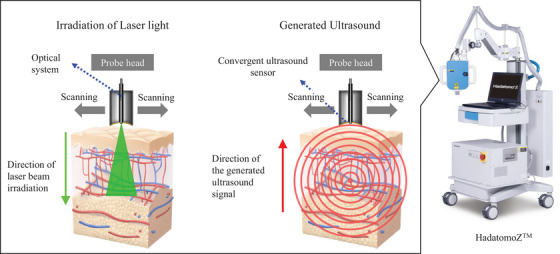
Photograph of Hadatomo Z, the photoacoustic measurement device used in this study, and schematic diagram of the measurement. The probe emits a laser as an incident light and receives an acoustic signal generated in the skin. The probe head scans to perform 3D measurements.

We used a Hadatomo Z WEL5200 (Advantest, Tokyo, Japan)^19^ PA measurement device as a base machine in this study (Figure 1). The system is capable of dual‐wavelength PA^20^ and ultrasound imaging. The center frequency of the ultrasound sensor is 60 MHz, enabling high resolution in‐vivo imaging. The original light sources for this machine were 532 and 556 nm laser unit or 575 and 650 nm laser unit, which are commercially available. The light source was changed for this study. In this study, we used two kinds of dual‐wavelength lasers: one for 575 and 586 nm and another one for 575 and 650 nm. The pulse energy is less than 18 μJ at 575 nm and less than 14 μJ at 650 nm in the 575 and 650 nm laser unit, less than 16 μJ at 575 and 586 nm in the 575 and 586 nm laser unit. The repetition rate is 1000 Hz and the pulse width is less than 10 ns for each. The solid‐state laser is used in the laser unit. The in‐house developed wavelength conversion element is installed to output each wavelength laser. Since the absorption coefficient of hemoglobin is high in the visible wavelength, in‐vivo PA signals can be obtained with low irradiation. The in‐house developed light source of 575 and 586 nm was used to measure the oxygen saturation value using the difference in absorption coefficients between oxy‐hemoglobin and deoxy‐hemoglobin. Since 650 nm represents melanin absorption, the clear blood vessel images can be obtained by subtracting 650‐nm PA signals from 575‐nm PA signals.^21^ The analyses of blood vessels and melanin were performed using subtracted and 650‐nm PA images, respectively. The measurement region was 9 × 9 mm with a 30‐μm scanning step along the vertical direction of the skin. The spatial resolution of the device was shown to be 24 μm in the horizontal direction and 16 μm in the vertical direction by a previous study that examined this device using a reference.^20^

### 2.4 Extraction of vascular and melanin structures from photoacoustic imaging data

The machine learning technique, Unet,^22^ was used to extract vascular and melanin structures from the PA images. Details of this extraction method were described in the supplemental data documentation. Please refer to that for those interested. The thickness, number of branches, and volume of the 3D structure were calculated as feature quantities for the “vascular structure” and “melanin three‐dimensional structure” in the extracted 3D data. The number of voxels of the extracted structure was counted and used as the volume. The extracted structure was then thinned and its direction vector was calculated. Cross‐sectional images orthogonal to the vector were obtained on a voxel basis. The radius was calculated from the cross‐sectional area, and the average of the radii was used as the thickness. The branches were counted using the Matlab branch search function on the thinned data. The depth of analysis for vascular structure was set for each participant to the depth at which the vascular structure could be visually identified on the image by the analyst. In the analysis, the 3D data were divided equally into three layers in the depth direction, and the first and second layers were used for the analysis from the surface layer. The third layer was excluded from the analysis due to its accuracy, because its deepest point was set visually by the analyst, as mentioned above.

### 2.5 Calculation of oxygen saturation

The spatial distribution of oxygen saturation, SO_2_(r), can be calculated as the ratio of oxygenated hemoglobin HbO_2_ to deoxygenated hemoglobin Hb as expressed by Equation (2).^23^

*r* represents positional information, ε_Hb_ is the molar absorption coefficient of deoxygenated hemoglobin, ε_ΔHb_ is the difference between the molar absorption coefficients of oxygenated and deoxygenated hemoglobin, and μ_a_(*r*) is the PA signal. Additionally, λ1 and λ2 are laser wavelengths, with λ_1_ and λ_2_ being 575 and 586 nm, respectively. On the assumption that the molar absorption coefficients of oxygenated and deoxygenated hemoglobin corresponding to wavelengths λ_1_ and λ_2_ are known values, the spatial distribution of oxygen saturation, SO_2_(r), can be calculated from the amplitude ratio of the PA signal at each wavelength, and this value was used in this study. Furthermore, using the same idea as in the analysis of the vascular structure, we divided it into three layers equally in the depth direction and used the first and second layers for the study.

### 2.6 Statistical analysis

All statistical analyses were conducted using the statistical software package JMP19 (SAS Institute, Cary, North Carolina, USA). Correlation analysis between measurements was performed using Spearman rank correlation coefficient analysis. Statistical significance of correlation coefficients was considered based on *p* < 0.05.

## 3 RESULTS

**FIGURE 2:**
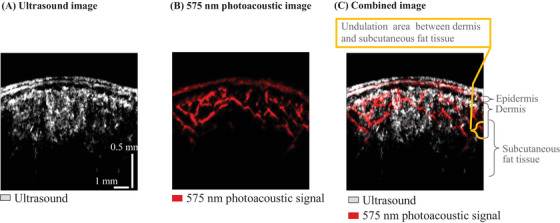
Typical image of photoacoustic measurement results combining ultrasound signals. (A) Ultrasound image. (B) 575‐nm photoacoustic image. (C) Combined image. In the ultrasound signal, findings with undulation structures are shown in the depth region indicated by the yellow line.

Figure 2 shows a representative example of PA measurements combining 575‐nm PA and ultrasound signals. Overlaying the PA image measuring the vascular structure, which is shown in red, with the ultrasound measurement image, which is shown as a white to black grayscale image, the vascular structure was observed down to the subcutaneous fat layer region that is deeper than the undulation region, which is the boundary structure between the dermis and subcutaneous fat layers.

**FIGURE 3:**
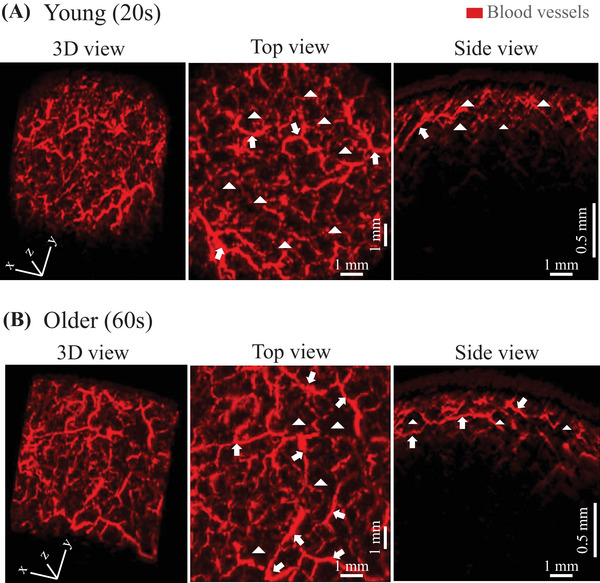
Typical results of vascular structure measurements. (A) Young (20s) participant. (B) Older participant (60s). In each case, the 3D view shows the entire measurement, the top view shows the result from the surface direction of the upper skin, and the side view shows the result from the horizontal cross‐sectional direction. Arrowheads in the figure indicate the presence or absence of fine vascular structures around the thick vascular structures. The younger participants show areas with fine vascular structures, whereas older participants show blank areas with no fine vascular structures. The arrows in the figure indicate thick and relatively continuous vessels.

**FIGURE 4:**
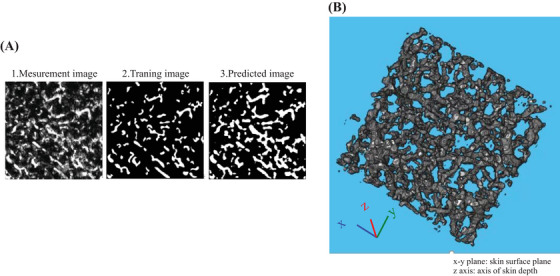
Results of vessel structure extraction performed using the constructed machine learning system. (A) Results of the verification against vessel extraction using a validation dataset that was not used for training. 1 is an arbitrary horizontal cross‐sectional image. 2 is an image in which the analyst manually extracted blood according to the visual impression of 1. 3 is an image of the vascular structure extracted by the machine learning system for 1. (B) Typical image of a 3D structure created by processing all *xy*‐planar parallel surfaces divided in the *z*‐axis direction using a machine learning system, and then merging them back together again.

**FIGURE 5:**
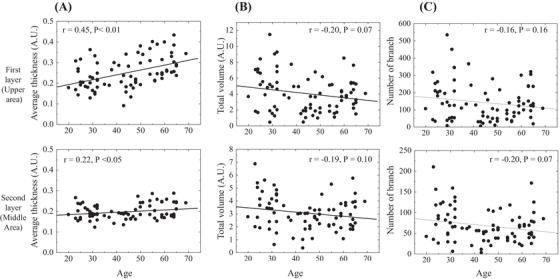
The extracted vascular structures are divided equally into three layers in the depth direction, and each feature of the structures in the first two layers from the top of the layers is calculated and plotted against age. (A) Average thickness of the vascular structure. (B) Total volume of the vascular structure. (C) Number of branches of the vascular structure.

The representative images of vascular structures extracted by subtracting 650‐nm PA signals from 575‐nm PA signals are shown in Figure 3A,B for young (20s) and old (60s) participants, respectively. In the comparison between the young and old participants, fine vascular structures were observed in the area indicated by the arrowheads in the “top view” in the young participants, whereas, in the old participants, there were blank areas without such structures. Contrarily, as indicated by the arrows, many continuous and relatively large vessels are observed in the elderly, participants whereas few vessels seem to have such characteristics in the young participants. The same tendency was observed in the images from Sideview. That is, while fine vessels were observed in the younger groups in relatively all layers measured, the older groups showed a predominance of large vessels, as well as the presence of areas without vessels with blackened out areas between them. Therefore, to quantify the individual differences and aging changes in blood vessel structure, we constructed a machine learning model and attempted to extract the blood vessel structure. Using the constructed training machine, the results of the confirmation for vessel extraction using a validation dataset that was not used for training are shown in Figure 4A. A representative example of a horizontal cross section (*xy*‐plane: plane parallel to the skin surface) from the PA imaging data is shown in Figure 4A1. The training image (Figure 4A2), which was created by the analyst by manually extracting the blood vessels for the target surface (Figure 4A1), and the predicted image (Figure 4A3) created by the training machine, showed a high homology. Using this machine learning model, the images processed for all *z*‐axis segmented planes were combined again in the *z*‐axis direction to generate a 3D structure. A representative example is shown in Figure 4B. The maximum measurement depth was set for each participant at a level at which the analyst could visually confirm the vascular structure. As a result, the depth was generally within 2 mm, as shown in Figure 2. For the extracted vascular structures, the measurement data were equally divided into three layers for each participant in the depth direction, and the feature values of the structures in the first two layers from the upper layer were calculated. The results are shown in Figure 5. The mean thickness of vascular structures were moderately and weakly negatively correlated with age for each depth. Although there was no statistically significant age‐related change in the volume or number of branches, there was a large dispersion of values in the younger group. The total volume of the first layer tended to be larger than that of the second layer. In fact, the average volume of the first layer for all ages was 1.3 times greater than that of the second layer.

**FIGURE 6:**
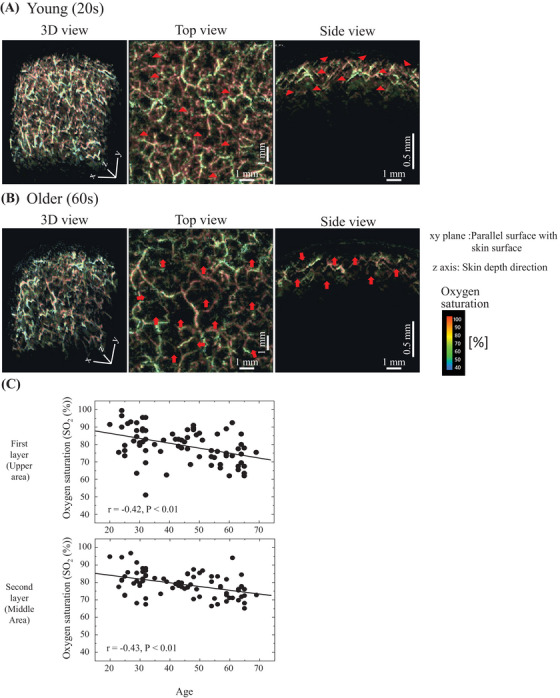
Typical results of oxygen saturation (SO2(r)) measurements. (A) Young (20s) participant. (B) Older participant (60s). In each case, the 3D view shows the entire measurement, the top view shows the result from the surface direction of the upper skin, and the side view shows the result from the horizontal cross‐sectional direction. Arrowheads in the figure indicate regions of high oxygen saturation. Moreover, arrowheads in the figure indicate blank areas where oxygen saturation is not detected. (C) The measurement data were divided equally into three layers in the direction of depth, and the oxygen saturation in the first two layers from the upper layer was calculated to evaluate aging changes.

In an examination of oxygen saturation (SO_2_(r)) using 575‐ and 586‐nm wavelengths, a spatial distribution similar to that of the vascular structure was observed (Figure 6). The typical measurement data for the young (20s) and old (60s) participants are shown in Figure 6A and B, respectively. In the top view, the younger group showed areas of high oxygen saturation in vessels with relatively fine structures, as indicated by the arrowheads. Contrarily, the older group tended to have missing areas of fine vessels with high oxygen saturation, as indicated by the arrow. This tendency is also seen in the side view, as indicated by the arrowheads, where regions of high oxygen saturation are seen throughout the vascular structure in the younger group, but no such regions were observed in the older group, resulting in a blank image, as indicated by the arrows. The layers were divided into three layers in the depth direction in the same way as the vascular structure, and the oxygen saturation SO_2_(r) was measured in the first and second layer of these layers (Figure 6C). The results showed that oxygen saturation tended to decrease with aging in both layers.

**FIGURE 7:**
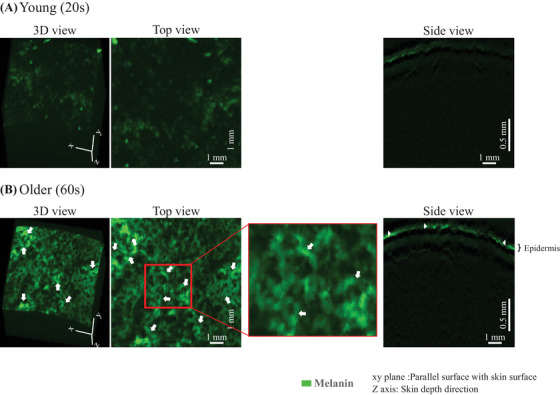
Typical example of measurement results of the state of melanin distribution in the skin. (A) Young (20s) participant. (B) Older participant (60s). In each case, the 3D view shows the entire measurement, the top view shows the result from the surface direction of the upper skin, and the side view shows the result from the horizontal cross‐sectional direction. The arrows in the figure indicate the torus‐like crest structure. Arrowheads also indicate areas in the extracted PA signal where the thickness can be visually confirmed.

**FIGURE 8:**
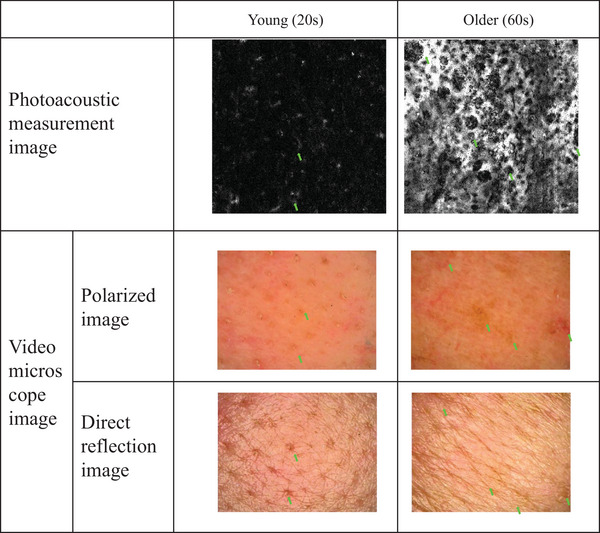
Comparison of photoacoustic and skin surface images acquired by using microscope at the same site. Typical examples of young and old groups. Arrows indicate the torus‐like pattern structures in the photoacoustic image. In the video microscopic image, the arrows indicate the same locations as in the photoacoustic image.

**FIGURE 9:**
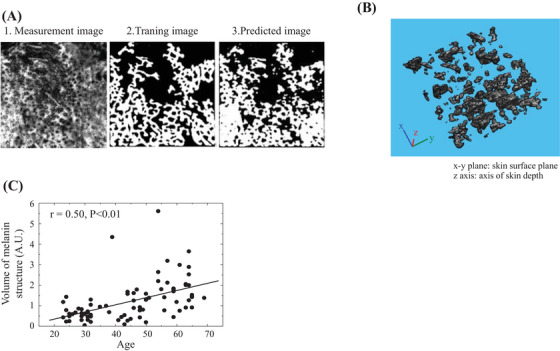
Results of melanin structure extraction performed using the constructed machine learning system. (A) Results of the verification against melanin structure extraction using a validation dataset that was not used for training. 1 is an arbitrary horizontal cross‐sectional image. 2 is an image in which the analyst manually extracted melanin structures according to the visual impression of 1. 3 is an image of the melanin structure extracted by the machine learning system for 1. (B) Typical image of a 3D structure created by processing all *xy*‐planar parallel surfaces divided in the *z*‐axis direction using a machine learning system, and then merging them back together again. (C) Age‐related changes in the volume of extracted melanin structures.

The state of melanin distribution was examined by using 650‐nm PA signals. The typical measurement results for the young (20s) and old (60s) individuals are shown in Figure 7A and B, respectively. The PA signal was less apparent in the younger group. Contrarily, the PA signal, shown in green, was clearly visible in the older group. Additionally, a torus‐like pattern structure was observed as indicated by the arrows in the figure (Figure 7B top view, enlarged). This structure also had a thickness, as indicated by the arrowheads in the side view. To confirm which phenotype of the appearance of the skin surface is related to the melanin features found by the PA method, a comparison was made using skin images acquired from the same area by video microscopy. Representative examples of younger and older groups are shown in Figure 8. The torus‐like structures measured by using the PA method, especially in the elderly, were found to coincide in site with the skin color irregularities around the pores that were not observed in the normal reflection video macroscopic images but observed in the polarized video microimages instead, as indicated by the arrows in the figure. The melanin structure was extracted. The results obtained using the constructed training machine with a dataset not used for training are shown in Figure 9. Compared to the annotation data (Figure 9A2), which were created by manually extracting melanin structures from the original image (Figure 9A1), the predicted image (Figure 9A3) extracted by the deep learning machine model showed a high homology. A representative example of the 3D results of melanin structures extracted using this method is shown in Figure 9B. The spatial structure of melanin was extracted. The volume of the 3D structure of the extracted melanin was analyzed, with the results shown in Figure 9C. It was shown that the volume of melanin structure increases with aging.

## 4 DISCUSSION

Using a PA method, noninvasive measurement of internal skin properties related to the vascular structure and oxygen saturation as well as 3D distribution of melanin in the facial cheek region was performed to analyze the individual differences and aging changes in these properties.

In the facial cheek, the dermis was reported to be about 1 mm thick.^24^ On the other hand, the boundary between the dermis and subcutaneous fat layers has an indistinct undulating structure,^25^ the measurement results of the previous study were considered to be measurements that included this structure. In the present PA measurement, it vascular structures can be measured even in the subcutaneous fat layer, which is the region deeper than 500 μm, where the undulation structure is observed (Figure 2). Previously, the vascular structure of the face has been measured using other noninvasive methods, such as OCT, which are limited to the dermal reticular layer at a depth of approximately 300 μm, the relatively shallow skin layer.^10^ Therefore, the usefulness of this method, which can be expected to analyze regions at greater depths, was demonstrated.

In the vascular structure, fine vessels were observed throughout the entire measured depth in the younger group, whereas, in the older group, these fine vessels were absent, and the areas without blackened vessels were observed in the corresponding regions (Figure 3). In this study, we constructed the machine learning model, and used it for structure extraction. The predicted vascular structures extracted by the constructed machine learning system showed a very high homology to the training images from which the analyst extracts the vessels based on the observations (Figure 4). Contrarily, the predicted images tend to be extracted slightly thicker than the training images and may not accurately reflect the thickness of the actual vascular structures. In the future, the training machine should be improved to extract images closer to the training image. Additionally, the measurement accuracy must be improved by eliminating distortions in the measurement image itself, so that the analyst can accurately extract structural quantities when training images are extracted. In addition to the abovementioned issues, the data may also contain distortions due to the effects of body movements during measurement. For these reasons, to understand the relative individual differences and aging changes, the units used in the calculation of vascular structure features were not expressed in the SI unit system, but were expressed in arbitrary unit (A.U.) based on pixel values. The measurement data were equally divided into three layers in the direction of depth, and structural features were calculated. As shown in Figure 2, the depth of analysis was approximately 2 mm. Therefore, the thickness per layer was approximately 0.6 mm. As seen in Figure 2, it was presumed that the first layer was almost the dermis layer, the second layer was the border region between the dermis and subcutaneous fat layers, and the third layer was the middle layer of the subcutaneous fat layer. The third layer was excluded from the analysis due to its accuracy, since the depth of the deepest section was set visually by the analyst in this study. The results of the feature analysis showed that the average thickness of vascular structures in the first and second layers became thicker with age; in other words, the fine vascular structures decreased, and this trend was more pronounced in the dermis layer than in the dermal–subcutaneous boundary layer. Although there was no statistically significant relationship between age and volume or number of branches, the dispersion of values was greater in the younger group. The total blood vessel volume was also larger in the dermal layer than in the dermal–subcutaneous boundary layer. As a limitation of this and other light‐based devices, the signal may decrease with depth. Also, it may reflect differences in anatomical superficial and deep vascular structures. Whether this result is due to the depth‐dependent characteristics, the device accuracy needs to be closely examined in the future through verification experiments using a phantom. These results indicate that the number of small blood vessels decreases with age, with this trend being stronger in the dermis layer, and that the total volume of blood vessels may be larger in the dermis layer, thereby suggesting that the characteristics of the vascular structure may differ between layers.

For the oxygen saturation (SO2(r)), the spatial distribution was visualized (Figure 6). Comparison of the data between the younger and older groups revealed a trend to a decreased high oxygen saturation state in the older group in areas where small blood vessels are expected to be present in the young group. Numerical results (Figure 6C) showed a tendency for oxygen saturation to decrease with aging in the upper and middle layers, with no significant differences in the depth direction between the two groups. Our previous NIRS studies have shown that oxygen saturation in all skin layers, from the superficial layer to the subcutaneous fat layer, decreases with age.^11^ The same trend was demonstrated in the present study.

Next, the melanin status in the skin was also investigated. As a result, a torus‐like pattern structure tended to increase with age (Figure 7). The extraction and analysis of this melanin structure showed that its volume increases with age (Figure 9C). This measurement excludes spots that can be seen visually, indicating that melanin may have structures that change with age even outside of the spot areas. In comparison with polarized video microscopic images of the same area, participants with such torus body structures showed color irregularities in the microscopic images (Figure 8). Therefore, it was speculated that this structure may be responsible for the uneven skin color appearance. Based on the contrast with the video microscopic image of the normal reflection, this torus structure probably matches the pore structure location. In principle, the spatial resolution of the PA method used in this study is approximately 30 μm, and melanin granules cannot be seen. Contrarily, although it is impossible to determine the granule‐level image, the high measurement sensitivity would have allowed capturing of the macroscopic structural features as the state of melanin presence in the space. Melanin has been found to be present not only in the epidermis but also in the dermis, as shown in Solar Lentigo lesions described previously.^26^, ^27^ A detailed study of how melanin is distributed in depth is needed in the future.

PA measurement has been reported to be influenced by skin color in various ways.^28^, ^29^ This study did not examine the relationship with skin tone. On the other hand, since only Japanese subjects from the same residential area were utilized, the results of this study are considered to have a certain degree of accuracy. Further investigation into the relationship between skin tone and the outcomes of this study will enable more accurate measurements. In this study, the same subject was measured once. Future studies with the same subjects should be conducted multiple times to verify the accuracy of the data, which would make the data more accurate and reliable.

## 5 CONCLUSION

PA measurements have revealed detailed properties of chromophores that may contribute to skin appearance, which has not been studied before, and have provided a new target for research on skin aging mechanisms. In the future, it is expected that this measurement method will be used more extensively to study physical properties in more detail.

## CONFLICT OF INTEREST STATEMENT

The authors declare that there is no conflict of interest that could be perceived as prejudicing the impartiality of the research reported.

## Supporting information

Supporting Information

Supporting Information

## Data Availability

The data that support the findings of this study are available on request from the corresponding author. The data are not publicly available due to privacy or ethical restrictions.
