# Supplementary material for: Quantitative analysis of age‐related changes in vascular structure, oxygen saturation, and epidermal melanin structure using photoacoustic methods
Source: Skin Res Technol. 2024 Jan 4;30(1):e13537. doi: 10.1111/srt.13537 (PMC10765365; doi:10.1111/srt.13537)
Supplement: Supplementary file 1 — Supporting Information [file SRT-30-e13537-s002.pptx]

## Slide 1
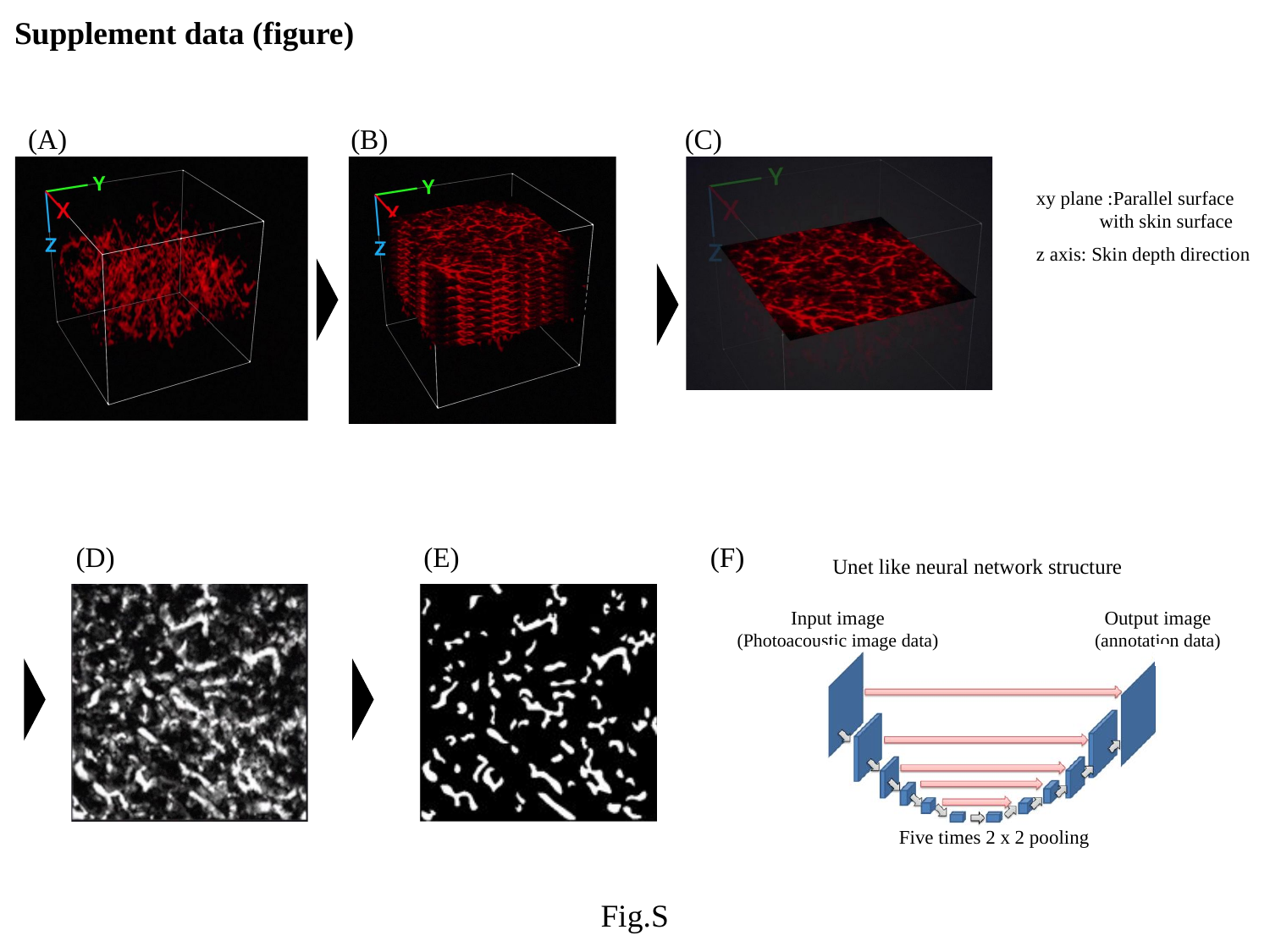

Supplement data (figure)
(A)
(B)
(C)
xy plane :Parallel surface
 with skin surface
z axis: Skin depth direction
(D)
(E)
(F)
Unet like neural network structure
Input image
(Photoacoustic image data)
Output image
(annotation data)
Five times 2 x 2 pooling
Fig.S
