# Supplementary material for: Quantitative analysis of age‐related changes in vascular structure, oxygen saturation, and epidermal melanin structure using photoacoustic methods
Source: Skin Res Technol. 2024 Jan 4;30(1):e13537. doi: 10.1111/srt.13537 (PMC10765365; doi:10.1111/srt.13537)
Supplement: Supplementary file 2 — Supporting Information [file SRT-30-e13537-s001.docx]

**Supplement data (manuscript)**

***Detail method for extraction of vascular and melanin structures from photoacoustic imaging data***

The machine learning technique, Unet^21^, was used to extract vascular and melanin structures from the photoacoustic images. The actual computational processing was performed using MATLAB® (MathWorks inc, Natick, USA) and TensorFlow, a module of Python 3.7. The analysis procedure is shown in Fig. S1, with the extraction of vascular structures as a representative example. The same procedure was used for the extraction of melanin structures. The 3D measurement data obtained by photoacoustic measurement (Fig. S1A) was reconstructed as stacked data to the depth direction (z-axis direction) of the horizontal plane (xy-plane) to the skin surface (Fig. S1B). Next, a number of xy-planes were randomly selected from the reconstructed data based on the depth direction (Fig. S1C). For the selected planar images (Fig. S1D), features that can be visually recognized as vascular structures by the analyst were traced and a binarized image were created (Fig. S1E). Ninety binarized images produced in this way were prepared from the data of several participants, and these were used as the annotation data for training to extract the vascular structure. The same method was used to produce annotation data in the extraction of melanin structures. Next, as a machine learning segmentation method, we constructed a network with an Encoder–Decoder model structure similar to that of Unet, as described below. The original xy-planar image (Fig. S1D in the case of blood vessels) was used as input image data, and a binary image (Fig. S1E in the case of blood vessels) created by the analyst for the same image as the input data was trained as annotation data to construct a machine learning system with five times 2 x 2 pooling (Fig. S1F). The objective function was set as the dice coefficients calculated by Equation 1 below. y is the pixel vector of the original xy-plane image, and $\tilde{\boldsymbol{y}}$ is the pixel vector of the annotation data. Our machine learning system can obtain a binarized extracted image of the vascular and melanin structures by inputting the xy-plane image.

Equation 1

$$f\left( \boldsymbol{y},\tilde{\boldsymbol{y}} \right)=\frac{2\boldsymbol{y}\cdot\tilde{\boldsymbol{y}}}{\sum\boldsymbol{y}+\sum\tilde{\boldsymbol{y}}}$$

The xy-planes from which the vascular composition or melanin structure was extracted using the machine learning model were combined again in the depth direction at the same interval as during the decomposition in the z direction to construct the 3D data. The thickness, number of branches, and volume of the 3D structure were calculated as feature quantities for the “vascular structure” and “melanin three-dimensional structure” in the constructed 3D data. The number of voxels of the extracted structure was counted and used as the volume. The extracted structure was then thinned and its direction vector was calculated. Cross-sectional images orthogonal to the vector were obtained on a voxel basis. The radius was calculated from the cross-sectional area, and the average of the radii was used as the thickness. The branches were counted using the Matlab branch search function on the thinned data. The depth of analysis for vascular structure was set for each participant to the depth at which the vascular structure could be visually identified on the image by the analyst. In the analysis, the 3D data were divided equally into three layers in the depth direction, and the first and second layers were used for the analysis from the surface layer. The third layer was excluded from the analysis due to its accuracy, because its deepest point was set visually by the analyst, as mentioned above.

**Figure S**

Diagram of data preparation for machine learning and construction of a machine learning system to extract vascular structures from photoacoustic measurement images.

(A) Photoacoustic 3D imaging data acquired.

(B) Stacked data sliced in the depth direction (z-axis direction) on a plane parallel to the skin surface (xy-plane) are created for the 3D data.

(C) Select an arbitrary layer.

(D) Gray-scaled image of th selected one layer, which is viewed vertically in the xy-plane.

(E) An image with regions that the analyst visually determines to be vascular structures extracted from the gray-scaled image. This image was used as annotation data.

(F) The original xy-plane image was used as the input image data, and training was performed using teacher data to construct a Unet-type machine learning system with five 2 x 2 poolings
